# An integrated transcriptome and expressed variant analysis of sepsis survival and death

**DOI:** 10.1186/s13073-014-0111-5

**Published:** 2014-11-26

**Authors:** Ephraim L Tsalik, Raymond J Langley, Darrell L Dinwiddie, Neil A Miller, Byunggil Yoo, Jennifer C van Velkinburgh, Laurie D Smith, Isabella Thiffault, Anja K Jaehne, Ashlee M Valente, Ricardo Henao, Xin Yuan, Seth W Glickman, Brandon J Rice, Micah T McClain, Lawrence Carin, G Ralph Corey, Geoffrey S Ginsburg, Charles B Cairns, Ronny M Otero, Vance G Fowler, Emanuel P Rivers, Christopher W Woods, Stephen F Kingsmore

**Affiliations:** Emergency Medicine Service, Durham Veterans Affairs Medical Center, Durham, North Carolina 27705 USA; Department of Medicine, Duke University Medical Center, Durham, NC 27710 USA; National Center for Genome Resources, Santa Fe, NM 87505 USA; Department of Immunology, Lovelace Respiratory Research Institute, Albuquerque, NM 87108 USA; Department of Pediatrics, Center for Translational Sciences, University of New Mexico, Albuquerque, NM 87131 USA; Center for Pediatric Genomic Medicine, Children’s Mercy Hospitals and Clinic, Kansas City, MO 64108 USA; Department of Emergency Medicine, Henry Ford Hospital, Detroit, Michigan 48202 USA; Department of Electrical & Computer Engineering, Duke University, Durham, NC 27710 USA; Department of Emergency Medicine, University of North Carolina School of Medicine, Chapel Hill, NC 27599 USA; Medicine Service, Durham Veterans Affairs Medical Center, Durham, NC 27705 USA; Department of Emergency Medicine, University of Michigan, Ann Arbor, MI 48109 USA

## Abstract

**Background:**

Sepsis, a leading cause of morbidity and mortality, is not a homogeneous disease but rather a syndrome encompassing many heterogeneous pathophysiologies. Patient factors including genetics predispose to poor outcomes, though current clinical characterizations fail to identify those at greatest risk of progression and mortality.

**Methods:**

The Community Acquired Pneumonia and Sepsis Outcome Diagnostic study enrolled 1,152 subjects with suspected sepsis. We sequenced peripheral blood RNA of 129 representative subjects with systemic inflammatory response syndrome (SIRS) or sepsis (SIRS due to infection), including 78 sepsis survivors and 28 sepsis non-survivors who had previously undergone plasma proteomic and metabolomic profiling. Gene expression differences were identified between sepsis survivors, sepsis non-survivors, and SIRS followed by gene enrichment pathway analysis. Expressed sequence variants were identified followed by testing for association with sepsis outcomes.

**Results:**

The expression of 338 genes differed between subjects with SIRS and those with sepsis, primarily reflecting immune activation in sepsis. Expression of 1,238 genes differed with sepsis outcome: non-survivors had lower expression of many immune function-related genes. Functional genetic variants associated with sepsis mortality were sought based on a common disease-rare variant hypothesis. *VPS9D1*, whose expression was increased in sepsis survivors, had a higher burden of missense variants in sepsis survivors. The presence of variants was associated with altered expression of 3,799 genes, primarily reflecting Golgi and endosome biology.

**Conclusions:**

The activation of immune response-related genes seen in sepsis survivors was muted in sepsis non-survivors. The association of sepsis survival with a robust immune response and the presence of missense variants in *VPS9D1* warrants replication and further functional studies.

**Trial registration:**

ClinicalTrials.gov NCT00258869. Registered on 23 November 2005.

**Electronic supplementary material:**

The online version of this article (doi:10.1186/s13073-014-0111-5) contains supplementary material, which is available to authorized users.

## Background

Sepsis is a heterogeneous syndrome that leads to significant morbidity and mortality. There are more than 750,000 cases per year in the United States [1] and up to 19 million cases per year worldwide [2]. Despite the availability of potent antibiotics and intensive care, mortality remains at 20% to 30% [1,3], accounting for up to 56% of all in-hospital deaths [4]. Moreover, the majority of in-hospital sepsis deaths occur in patients with mild clinical disease that would not warrant early goal-directed therapy [4]. That mild initial clinical illness progresses to severe sepsis and death despite appropriate clinical care highlights host responses to sepsis that differ between survivors and non-survivors. Even among survivors, there remains a high rate of morbidity and mortality after hospital discharge identifying another unmet prognostic need [5].

In 1992, an international consensus conference defined sepsis as the systemic inflammatory response (SIRS) to the presence of infection [6]. Standardizing this definition enabled providers to rapidly identify and treat the condition. It also facilitated research with improved dissemination and application of information. However, the simplicity of this definition masks the tremendous complexity of the condition. Sepsis is not a single disease, but rather a highly heterogeneous syndrome that is the net result of host and pathogen interactions triggering networks of biochemical mediators and inflammatory cascades in multiple organ systems. It is influenced by many variables including pathogen, site of infection, clinical interventions, host genetics, age, and baseline health. As such, therapeutic trials have been largely disappointing in part because a one-size-fits-all approach fails to recognize the heterogeneity among patients with sepsis. This has stifled sepsis clinical research as evidenced by the small number of sepsis-focused clinical trials, comprising only 3% of all infectious disease-related research registered in ClinicalTrials.gov [7]. However, interventions considered failures may in actuality be highly effective in selected subpopulations. Understanding the spectrum of sepsis pathophysiology in a heterogeneous human patient population is a necessary first step to redefining this syndrome and individualizing sepsis management [8].

We previously performed comprehensive, integrated analyses of clinical and molecular measurements in sepsis to identify and prioritize sepsis pathways in survivors and non-survivors without the bias of *a priori* mechanistic hypotheses [9-13]. This included the derivation of a signature, derived from clinical, metabolome, and proteome data, that differentiated sepsis from SIRS of other etiologies and improved the prediction of survival and death in patients with sepsis [11]. Moreover, the proteome and metabolome were similar in survivors regardless of initial sepsis severity, and yet uniquely different from non-survivors, generating the hypothesis that initial host molecular response is a superior prognostic indicator compared to clinical staging criteria. Here, in a final orthogonal analysis, we sought unbiased associations with peripheral blood transcription and expressed nucleotide variants. We again hypothesized that an agnostic systems biology approach would reveal important biological associations informing sepsis diagnosis and prognosis. This analysis revealed many pathways as relevant to sepsis diagnosis, particularly immune activation: Both SIRS and sepsis non-survivors had lower gene expression levels across multiple immune activation pathways. An additional hypothesis was that the transcriptome included expressed sequence variants associated with sepsis outcome under the common disease-rare variant premise. Indeed, we observed the presence of expressed sequence variants in *VPS9D1* to be associated with sepsis survival. However, no associations with mitochondrial gene variants were identified despite previous observations that mitochondrial biology is important for sepsis outcomes. These results highlight the complex role of immune function in sepsis, indicating differences between survivors and non-survivors. Moreover, we identified genetic variants associated with sepsis outcome. Their discovery offers a potential explanation for the underlying heterogeneity behind sepsis outcomes that often confounds available clinical prognostic tools.

## Methods

### Patient selection and clinical data collection

The CAPSOD study was approved by the Institutional Review Boards of the National Center for Genome Resources, Duke University Medical Center, Durham Veterans Affairs Medical Center and Henry Ford Health Systems and filed at ClinicalTrials.gov (NCT00258869). This research conformed to the Helsinki Declaration. Inclusion criteria were presentation of adults at the ED with known or suspected acute infection and presence of at least two SIRS criteria (tympanic temperature <36°C or >38°C, tachycardia >90 beats per minute, tachypnea >20 breaths per minute or PaCO_2_ <32 mmHg, white cell count <4,000 cells/mm^3^ or >12,000 cells/mm^3^ or >10% neutrophil band forms) [10,12,13]. Exclusion criteria were as previously described [10,12,13]. Patients were enrolled from 2005 through 2009 and written informed consent was obtained by all study participants or their legal designates. Adults aged 17 years or older were included for this analysis.

Patient demographics, past medical history, physical examination, and APACHE II were recorded at enrollment using online electronic data capture (Prosanos Inc., Harrisburg, PA, USA) [10,12-15]. Microbiologic evaluation was as clinically indicated and in some cases was supplemented by multiplex PCR to identify bloodstream infections (The LightCycler® SeptiFast M GRADE Test, Version 2.0; Roche, Basel, Switzerland) [13].

All subject records were adjudicated at least 28 days after enrollment by a physician with emergency medicine training (SWG) to determine whether presenting symptoms and signs were due to infection, etiologic agent, site of infection, patient outcome, and time to outcome [10,13]. A second physician with infectious diseases training (ELT) independently adjudicated a 10% sample, selected at random. Agreement regarding infection classification was high with κ = 0.82, exceeding the 0.80 threshold considered ‘almost perfect agreement’ [10,16]. All adjudications were performed prior to the generation of any transcriptome data.

Subjects were classified into one of five groups that reflected the conventional concept of sepsis progression as a pyramid [1,4]: (1) Uncomplicated sepsis (sepsis without disease progression); (2) Severe sepsis (severe sepsis at t_0_ or progression to severe sepsis by day 3); (3) Septic shock (septic shock at t_0_ or progression to septic shock by day 3); (4) Sepsis non-survivors (sepsis of any severity at the time of enrollment and death within 28 days); and (5) SIRS (≥2 SIRS criteria without evidence of infection). Based on experimental results presented here, it was determined that the sepsis survivors (uncomplicated sepsis, severe sepsis, and septic shock) had similar transcriptional profiles. Consequently, they were recoded as a single ‘sepsis survivor’ group.

CAPSOD was designed to support a variety of research questions. Therefore, although 1,152 subjects had enrolled in CAPSOD by the time of this analysis, 129 subjects were chosen for the work presented here. This number was based on several factors. First, these samples were matched to metabolomic and proteomic data [11], where a sample size of 30 subjects in each of the five groups was calculated to provide 80% power to test associations with survival/death. Although the initially selected group consisted of 150 subjects, subjects were excluded from transcriptome and expressed sequence variant analysis due to lack of PAXgene RNA tubes, insufficient RNA, or poor quality RNA. The final number of subjects per group was 28 sepsis non-survivors, 23 SIRS survivors, and 78 sepsis survivors.

### Sample collection and preparation

Blood collections occurred at t_0_, corresponding to the day of enrollment upon presentation to the ED. Whole blood was collected in PAXgene RNA tubes (Qiagen, CA, USA) to stabilize intracellular RNA and subsequently stored at −80°C until use. RNA was prepared using a PaxGene Blood RNA kit (Qiagen) according to the manufacturer’s instructions. Nucleic acids were pelleted by centrifugation, washed, and treated with proteinase K. Residual cell debris was removed by centrifugation through a column. Samples were equilibrated with ethanol and total RNA was isolated using a silica membrane. Following washing and DNase I treatment, RNA was eluted. RNA integrity was determined by 2100 Bioanalyzer microfluids using RNA 600 Nano kit (Agilent), averaging 7.6 (standard deviation 1.7). RNA samples were stored at −80°C.

### RNA sequencing

mRNA sequencing libraries were prepared from total RNA using the Illumina mRNA-Seq Sample Prep Kit (Illumina, catalog # RS‐100‐0801), according to the manufacturer’s recommended protocols and as we have previously published [17]. Briefly, mRNA was isolated using oligo-dT magnetic Dynabeads (Invitrogen). Random-primed cDNA was synthesized and fragments were 3’ adenylated. Illumina DNA oligonucleotide sequencing adapters were ligated and 350 to 500 bp fragments were selected by gel electrophoresis. cDNA sequencing libraries were amplified by 18 cycles of PCR and quality was assessed with Bioanalyzer. cDNA libraries were stored at −20°C.

CAPSOD experimental samples were sequenced without multiplexing on Illumina GA_*IIx*_ instruments (54-cycle singleton reads). This yielded 13.4 million reads, totaling 718.4 Mbp of sequence, and nine-fold average coverage. Base calling was performed using Illumina Pipeline software v1.4, except for 14 samples performed with v1.3. Approximately 500 million high quality reads were generated per sample. Data can be accessed via the Gene Expression Omnibus repository (GSE63042).

Sequence quality analysis was performed on the raw data using FastQC version 0.10.1, assessing per-base and overall sequence quality, nucleotide composition, and uncalled bases. Quality trimming and adapter clipping were performed using Trimmomatic version 0.32, trimming trailing bases below Phred quality score of 20 (which corresponds to a 99% base call accuracy rate), and discarding clipped reads shorter than 25 bp. FastQC was used to re-assess the integrity of the clipped reads prior to subsequent mapping and analysis. On average, over 93% of the sequences had a mean Phred base call quality of 20 or higher after trimming. The post-trimming uncalled base rate was 0.09%. The Illumina iGenomes UCSC hg19 human reference genome and annotation was used as a reference, downloaded March 2013. Clipped reads were mapped to the hg19 genome using Tophat version 2.0.7, and assembled with Cufflinks version 2.0.2, all with default parameter settings. The average mapping rate was 77.7%. Read counts for each gene were obtained with HTSeq version 0.5.4, specifically the intersection-nonempty mode of htseq-count. SAM/BAM conversions, sorting, indexing, and marking of PCR duplicates were performed with SAMtools version 0.1.18 and Picard version 1.83.

For variant analysis, sequence data were aligned to the GRCh37.p5 human reference genome using STAR [18]. Read alignments were processed with the Genome Analysis Tool Kit [19] (GATK) version 3.1. Duplicate reads were removed and single nucleotide polymorphisms (SNP) and insertion/deletion (INDEL) discovery and genotyping was performed on all samples individually using the GATK HaplotypeCaller producing a standard variant call format (VCF) [20]. Resulting nuclear variants were hard filtered to keep variants with a Phred scaled quality score of 20 or higher (a measure of quality of DNA sequence) [21,22]. To address issues with varying coverage in the mitochondrial genome, samples were filtered so that only 91 samples with at least 85% of the mitochondrial genome covered by 16 reads or more were included in the final variant analysis. Further, mitochondrial variants were only analyzed if they were identified in 10 reads or more.

Variants were annotated with the Rapid Understanding of Nucleotide variant Effect Software (RUNES v1.0) [23]. RUNES incorporates data from ENSEMBL’s Variant Effect Predictor software [24], and produces comparisons to NCBI dbSNP, known disease mutations from the Human Gene Mutation Database [25], and performs additional *in silico* prediction of variant consequences using RefSeq and ENSEMBL gene annotations. RUNES categorizes each variant according to American College of Medical Genetics and Genomics recommendations for reporting sequence variation [7,8] as well as an allele frequency derived from the Children’s Mercy Hospital Center for Pediatric Genomic Medicine Variant Warehouse database [23]. As multiple transcripts exist for *VPS9D1*, the locations of each variant with respect to the cDNA and protein for each identified transcript are presented in Additional file 1.

### Statistical analyses

Overlaid kernel density estimates, Mahalanobis distances, univariate distribution results, correlation coefficients of pair wise sample comparisons, unsupervised principal components analysis (by Pearson product–moment correlation), and Ward hierarchal clustering of Pearson product–moment correlations were performed using log_2_-transformed data as described [17] using JMP Genomics 6.1 (SAS Institute). ANOVA was performed between sepsis groups, with a 7.5% FDR correction based on the Storey method [17,26,27]. FDR calculations used for all other analyses employed the Benjamini-Hochberg method [28]. ANOVA was also performed for *VPS9D1* variants in the sepsis survivors and non-survivors. The patients were separated based on whether they had the expressed variant or not. Subjects without adequate sequencing coverage across the variant were excluded from the analysis. Pathway gene list enrichment analysis was performed using the ToppFun algorithm of the ToppGene Suite [29].

VCF files for sepsis survivors and non-survivors were analyzed using the SNP and Variation Suite v8.1.4 (GoldenHelix). To assess the association of genetic variation with sepsis outcomes we conducted three separate analyses of two groupings of detected variants. The groupings of variants were: (1) all variants within 5 kb of annotated genes; and 2) only variants likely to have a functional impact by limiting to non-synonymous, in/del, and frameshift variants in exons as identified using RefSeq 63 (v. 2014-02-16). We first examined the presence or absence of variants within a gene and its association with sepsis outcomes using a Fisher’s Exact Test for Binary Predictors (Fisher’s binary). Associations were also sought between the total number of variants per gene and sepsis non-survival by correlation, t-test, and regression analysis. For rare variant analysis we used the Combined Multivariate and Collapsing method and Hotelling T Squared Test with a minor allele frequency bin of <0.01 [30]. To create the allele frequency bins for grouping 1 we used the 1 k genome all populations MAF [31] and for grouping 2 we used the NHLBI exome variant server all populations MAF [32].

## Results

### Study design and clinical synopsis

**Figure 1:**
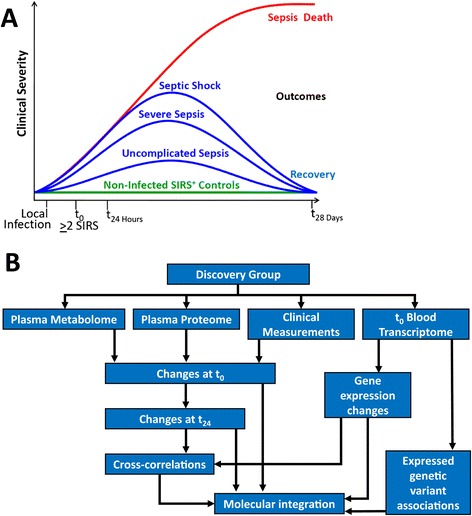
**A systems survey of sepsis survival. (A)** Schematic representing the different trajectories enrolled subjects might take. X-axis represents time (not to scale), emphasizing the illness progresses from local to systemic infection prior to clinical presentation (t_0_). The green line is flat only to distinguish subjects without infection, although these individuals could also have the full spectrum of clinical illness severity. Blue lines represent subjects with sepsis of different severities, all of whom survive at 28 days. This is in contrast to subjects with sepsis who die within 28 days, independent of initial sepsis severity. **(B)** Analytical plan for the CAPSOD cohort including previously published metabolome and proteome [11]. Metabolomic and proteomic analyses were performed on samples obtained at t_0_ and 24 h later. Transcriptomic analysis was performed on samples obtained at t_0_.

**Figure 2:**
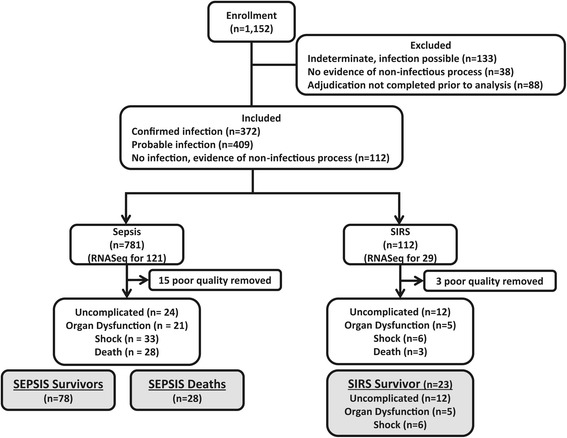
**CONSORT flow chart of patient enrollment and selection.** The planned study design was to analyze 30 subjects each with uncomplicated sepsis, severe sepsis (sepsis with organ dysfunction), septic shock, sepsis deaths, and SIRS (no infection present). However, limited sample quality or quantity in some cases decreased the number available per group. The analysis population includes 78 sepsis survivors, 28 sepsis non-survivors, and 23 SIRS survivors. Three SIRS non-survivors represented too few subjects to define their own analysis subgroup and were therefore removed prior to analysis.

The Community Acquired Pneumonia and Sepsis Outcome Diagnostics (CAPSOD) study was an observational trial enrolling subjects with community-acquired sepsis or pneumonia (ClinicalTrials.gov NCT00258869) (Figure 1A). Its focus was to define sepsis biology and to identify diagnostic and prognostic biomarkers in sepsis utilizing comprehensive clinical information and bioinformatic, metabolomic, proteomic, and mRNA sequencing technologies (Figure 1B). Subjects with suspected sepsis were enrolled in the emergency departments of Henry Ford Health System (Detroit, MI, USA), Duke University Medical Center (Durham, NC, USA), and the Durham Veterans Affairs Medical Center (Durham, NC, USA) from 2005 to 2009 by which time 1,152 subjects were enrolled [10-13] (Figure 2). Some enrolled subjects were later determined not to have sepsis, but rather a non-infectious systemic inflammatory response syndrome (SIRS). Infection status and 28-day mortality were independently adjudicated by a board-certified clinician followed by a second, confirmatory adjudication of 10% of cases (κ = 0.82) as previously described [10,12,13]. An indeterminate infection status in 259 subjects led to their exclusion (Figure 2). Twenty-eight day mortality in the remaining population of 893 was low (5.9%). Five subgroups were selected for mRNA sequencing: (1) Uncomplicated sepsis (n = 24); (2) Progression to severe sepsis within 3 days (n = 21); (3) Progression to septic shock within 3 days (n = 33); (4) Sepsis non-survivors at 28 days (n = 28); and (5) Patients with SIRS (n = 23). Subjects for each group were chosen to match non-survivors based on age, gender, race, enrollment site, and microbiological etiology (Table 1). As CAPSOD was an observational study, clinical care was not standardized and was determined by individual providers. Moreover, treatment administered to patients prior to enrollment (for example, self-administered, prescribed by outpatient providers, given by emergency medical services, or given in the ED) were not recorded and therefore were not controlled for in subsequent analyses.

**Table 1:** **Clinical and demographic information for the analysis population**

**Clinical variable**	**SIRS**	**Sepsis survivors**	**Sepsis non-survivors**
n	23	78	28
Age (years)	64.9 ± 14.4	56.1 ± 18.0	67.6 ± 17.0
Gender (% Male)	34.8%	59.0%	60.7%
Race (B/W/O)	16/6/1	47/26/5	21/6/1
APACHE II	16.8 ± 7.7	14.7 ± 6.6	21.3 ± 7.1
Pathogen^a^			
*S. aureus*	N/A	20 (26%)	5 (18%)
*S. pneumoniae*	N/A	20 (26%)	4 (14%)
Enterobacteriaceae	N/A	23 (29%)	3 (11%)
Total leukocyte count^b^	11.2 (8.8, 13.5)	14.6 (9.7, 18.7)	15.1 (10.4, 21.9)
% Neutrophils	77.0 (73.5, 83.3)	85.0 (82.0, 91.0)	87.4 (82.0, 92.8)
% Lymphocytes	13.0 (7.6, 15.8)	7.0 (4.0, 11.0)	8.0 (4.2, 11.8)
% Monocytes	7.1 (4.4, 9.8)	5.0 (3.0, 8.0)	4.5 (2.0, 6.0)
Co-morbidities			
Alcohol abuse	17.4%	17.9%	10.7%
Neoplastic disease	13.0%	6.4%	21.4%
Diabetes	30.4%	32.1%	35.7%
Congestive heart failure	0%	6.4%	14.3%
Chronic kidney disease	26.1%	21.8%	25.0%
Chronic liver disease	8.7%	5.1%	21.4%
Immunosuppression	0%	6.4%	7.1%
Smoker	21.7%	30.8%	25.0%

Data presented as mean ± standard deviation. ^a^Other identified pathogens include: *Candida albicans*, *Clostridium difficile*, Coagulase-negative Staphylococcus, *Enterococcus* species, Legionella, *Listeria monocytogenes*, *Mycoplasma pneumoniae*, *Pseudomonas aeruginosa*, Streptococcus non-pneumoniae (*agalactiae*, *pyogenes*, viridans group). No significant differences in pathogen frequency were identified between Sepsis Survivors and Sepsis Non-survivors using Fisher’s exact test. Subjects were counted more than once in cases of polymicrobial infection.

^b^Reported as cells x 10^9^/liter, median (1st quartile, 3rd quartile). Leukocyte differential percentages exclude one SIRS subject, nine Sepsis Survivors, and two Sepsis Deaths for whom differential data were not available.

B/W/O: black/white/other; N/A: not applicable.

### Peripheral blood gene expression analysis

Transcription in venous blood of patients at ED arrival was evaluated by sequencing of stabilized mRNA, which was chosen for its dynamic range, excellent correlation to qPCR, and capture of *in vivo* transcription early in sepsis evolution [33]. Furthermore, RNAseq permits the identification of expressed nucleotide variants, providing an opportunity to study genetic variation associated with phenotypes of interest [34-36]. Leukocyte number and differential cell counts were similar across groups (Table 1). mRNA sequencing for 129 subjects to an average depth of 13.5 million reads/sample yielded relative levels of transcription of 30,792 genes (of which 18,078 mRNAs were detected in >50% of subjects). Similar to the proteome and metabolome [11], ANOVA did not find any significant differences in gene expression between uncomplicated sepsis, severe sepsis, and septic shock groups, which consequently combined to form the ‘Sepsis Survivor’ group. This created three groups for comparison: Sepsis Survivor (n = 78), Sepsis Non-survivor (n = 28), and SIRS control (n = 23), as had been utilized for prior metabolomic and proteomic analyses [11].

**Figure 3:**
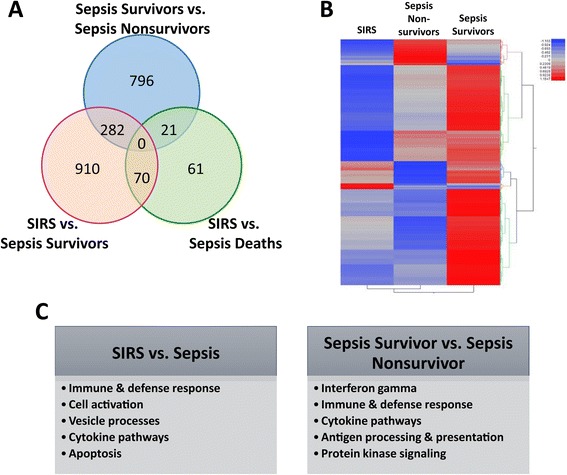
**Differentially expressed genes and pathways. (A)** Number and overlap among the differentially expressed, annotated genes in each pairwise comparison. **(B)** Hierarchical clustering of 2,140 differentially expressed gene (including 314 unannotated loci) using Pearson’s moment correlations applied to subjects with SIRS, Sepsis Non-survivors, and Sepsis Survivors. ANOVA with 7.5% FDR correction; −log10 *P* value = 2.21. **(C)** Highly represented ToppGene pathways and processes among the annotated genes differentially expressed between SIRS and Sepsis Survivors as well as Sepsis Survivors and Sepsis Non-survivors.

Differences in transcript abundance were measured between groups. There were 2,455 significant differences between all pairwise comparisons (Figure 3 and Additional file 2) based on ANOVA with a 7.5% false discovery rate (FDR), chosen to impart a greater degree of specificity. These 2,455 expression differences included 315 unannotated loci. The number of genes in each pairwise comparison is depicted in Figure 3A along with an expression heat map in Figure 3B. The first focus was to distinguish sepsis from SIRS, which is a particularly important diagnostic decision made at a patient’s first clinical contact. We therefore combined all sepsis survivors and sepsis non-survivors to create a Sepsis category, which was then compared to SIRS. There were 338 genes with significantly different expression, the majority of which (317/338; 94%) were upregulated in subjects with sepsis, indicating a robust increase in gene expression. Gene enrichment and pathway analysis was performed with the ToppFun algorithm [29]. The highly significant pathways differentiating sepsis and SIRS included response to wounding, defense response, and the immune or inflammatory response. Among the genes downregulated in sepsis, there were few significant pathways. One notable example of decreased gene expression in sepsis was *PROC* (Protein C), a key regulator of fibrin clot formation [37,38]. This plasma protein, often depleted in severe sepsis, was the basis for recombinant activated protein C as the only drug approved for the treatment of severe sepsis. Subsequent trials failed to replicate the beneficial effects, prompting its removal from the market [39]. *PROC* expression was decreased to a similar degree in sepsis survivors and sepsis non-survivors when compared to SIRS.

Prior metabolomic and proteomic studies suggested broad differences exist in the biochemistry of sepsis survivors and non-survivors. As such, differential gene expression and pathway analysis was repeated, focusing only on sepsis survivors as compared to SIRS (all of whom survived in the analysis population). This identified 1,358 differentially expressed genes, of which 1,262 were annotated. As before, the majority were increased in sepsis (1,317/1,358; 97%). Pathway analysis revealed similar results to the comparison of all sepsis and SIRS including immune-related categories such as immune response, defense response, response to wounding, and innate immune response (Figure 3C and Additional file 3). The increased expression of immune function-related pathways is consistent with the host need to combat infection. Moreover, subjects in this sepsis cohort were categorized by the type of pathogen: Gram positive or Gram negative (Table 1). A comparison of gene expression in these groups revealed that no genes met the cutoff for statistical significance, recapitulating the plasma proteomic and metabolomic findings in this comparison [11].

Among subjects with sepsis, another important clinical challenge is distinguishing those who will respond to standard treatment from those at highest risk of sepsis progression and mortality. We therefore focused on the 1,238 genes differentially expressed (1,099 annotated) between sepsis survivors and sepsis non-survivors. The majority (1,113/1,238; 90%) showed increased expression in sepsis survivors (Additional file 2). Pathway analysis revealed similar findings to the comparison of SIRS and sepsis. Specifically, sepsis survivors had increased expression of genes involved in the immune response including response to interferon-gamma, the defense response, and the innate immune response (Figure 3C and Additional file 3). Despite the infectious etiology of their illness, sepsis non-survivors had a muted immune response as measured by peripheral blood gene expression. Although the difference in total leukocyte count approached statistical significance (*P* value 0.06 by t-test), the differential cell count was similar between survivors and non-survivors (*P* value 0.56 for % neutrophils by t-test) (Table 1).

### Genetic associations with sepsis outcome

We next sought genetic associations with sepsis outcomes that might underpin the proteomic, metabolomic, and transcription changes in the CAPSOD cohort, potentially providing a unifying mechanism of sepsis death or survival. Genotypes were determined at each nucleotide in the expressed mRNA sequences of the 78 sepsis survivors and 28 sepsis non-survivors (homozygous reference, heterozygous variant, homozygous variant, not called).

Genetic associations were initially sought between sepsis outcome and mRNA variants of all types and allele frequencies mapping within 5 kb of an exon. These criteria were met by 417,570 variants in 18,303 genes. To narrow this number, three methods were utilized. The first collapsed heterozygous and homozygous variants in each gene, and scored binary associations of variant-associated genes with the sepsis outcome groups using the numeric Fisher’s Exact Test for Binary Predictors (Fisher’s binary). Second, associations were sought between the number of variants per gene and sepsis non-survival by correlation, t-test, and regression analysis. Finally, the Combined Multivariate and Collapsing method and Hotelling T Squared Test were applied [30]. No significant gene associations with sepsis outcome were found (FDR <0.10).

**Figure 4:**
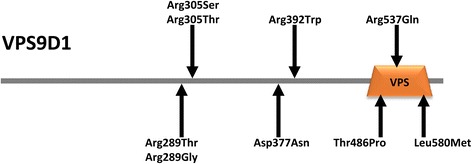
**Protein structure of VPS9D1 showing approximate location of variants associated with sepsis survival.**

We then looked for associations between sepsis outcome and mRNA variants likely to have functional effects, specifically 20,168 potentially phenotype-causing variants mapping to 6,793 coding domains. Our hypothesis was that common metabolomic, proteomic, or transcriptional phenotypes of sepsis non-survival might be causally related to multiple rare variants on a gene-by-gene basis. One gene, Vacuolar Protein Sorting 9 Domain-containing gene 1 (*VPS9D1*), showed significant associations between potentially functional mRNA variants and sepsis survival (Figure 4).

*VPS9D1* (transcript NM_004913) variants were significantly associated with sepsis outcomes as measured by Fisher’s binary (−log_10_*P* value 4.48, FDR = 0.07, odds ratio 0.08) and regression (−log_10_*P* value 5.03, FDR = 0.01, odds ratio 0.09). After excluding subjects with inadequate sequence coverage, nine unique non-synonymous substitutions were identified. Since any given subject could have more than one of these unique variants, we identified 46 variants in 36 subjects (Table 2). Forty-four *VPS9D1* variants were identified in sepsis survivors and two variants in sepsis non-survivors. Of the nine variants, the A > C substitution at chr16:89775776 (NC_000016.9 (GRCh37.p13) g. 89775776 A > C; NM_004913.2:c.1456A > C; NP_004904.2: p.Thr486Pro) occurred most commonly in the CAPSOD cohort. It was heterozygous in two of 26 (7.7%) sepsis non-survivors compared to 30 of 74 (40.5%) sepsis survivors (Table 2). The remaining eight non-synonymous variants were found less frequently, each occurring in two or fewer subjects and only in the sepsis survivor group. Seven variants were very rare (minor allele frequency, MAF <0.002) and two were rare (MAF <0.02). Although expression of *VPS9D1* was significantly decreased in sepsis non-survivors*,* this did not markedly decrease the number of comparisons between nucleotide variants and sepsis outcomes.

**Table 2:** **Expressed sequence variants identified in** ***VPS9D1***

**Chromosome (Start:Stop)**	**Variant type**	**Reference allele**	**Variant allele**	**cDNA change**	**Protein change**	**Variant impact**	**Reference SNP ID**	**Sepsis non-survivors**	**Sepsis survivors**
16 (89774899:89774899)	Substitution	G	T	c.1738C > A	p.Leu580Met	Non-synonymous	rs182342705	0/20	0/69
16 (89775352:89775352)	Substitution	C	T	c.1610G > A	p.Arg537Gln	Non-synonymous		0/6	1/27
16 (89775776:89775776)	Substitution	T	G	c.1456A > C	p.Thr486Pro	Non-synonymous		2/26	30/74
16 (89777078:89777078)	Substitution	G	A	c.1174C > T	p.Arg392Trp	Non-synonymous	rs56288641	0/20	2/67
16 (89777123:89777123)	Substitution	C	T	c.1129G > A	p.Asp377Asn	Non-synonymous	rs148694296	0/23	1/68
16 (89777306:89777306)	Substitution	G	T	c.946C > A	p.Pro316Thr	Non-synonymous		0/25	2/76
16 (89777337:89777337)	Substitution	T	A	c.915A > T	p.Arg305Ser	Non-synonymous		0/15	2/67
16 (89777338:89777338)	Substitution	C	G	c.914G > C	p.Arg305Thr	Non-synonymous		0/16	2/66
16 (89777386:89777386)	Substitution	C	G	c.866G > C	p.Arg289Thr	Non-synonymous		0/23	2/74
16 (89777387:89777387)	Substitution	T	C	c.865A > G	p.Arg289Gly	Non-synonymous		0/23	2/74

A given subject may harbor more than one variant. Multiple transcripts and corresponding proteins exist for *VPS9D1.* cDNA and protein changes are based on *VPS9D1* transcript NM_004913.2 and protein NP_004904.2.

**Figure 5:**
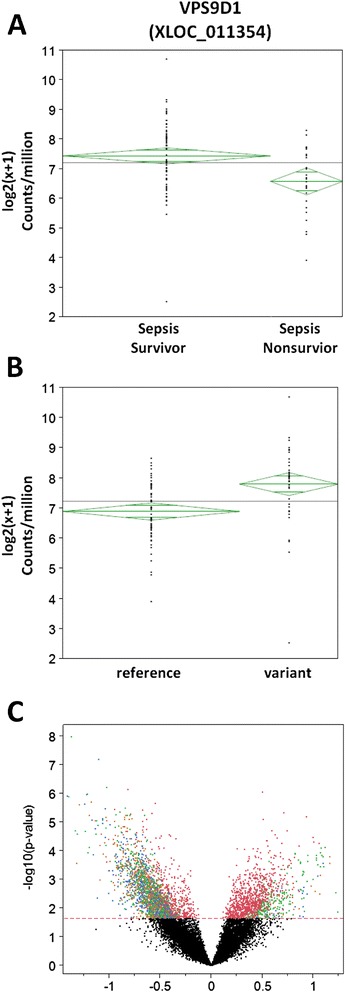
**Expression of** ***VPS9D1*** **.** *VPS9D1* is represented by two different genetic loci: XLOC_011354 (Cufflinks Transcript ID TCONS_00032132; RefSeq ID NM_004913) and XLOC_010886 (Cufflinks Transcript ID TCONS_00030416; RefSeq ID NM_004913). The former demonstrated greater sequencing coverage and is presented here. Results for XLOC_010886 were similar (data not shown). **(A)** Level of *VPS9D1* expression in sepsis survivors (n = 74) and sepsis non-survivors (n = 26). **(B)** Level of *VPS9D1* expression as a function of the *VPS9D1* reference (n = 64) or variant sequence (n = 36) among subjects with adequate coverage. **(C)** Volcano plot depicting differentially expressed genes as a function of the *VPS9D1* reference or variant allele.

The biological consequences of these variants are unknown. To determine if these variants were associated with gene expression changes, we defined two new analysis populations: subjects with and without a variant in *VPS9D1*. Genes with differential expression in these groups were identified followed by pathway analysis. Individuals with variants in *VPS9D1* differed in expression of 3,799 genes, representing many different pathways (Figure 5; Additional file 4). Among the most highly significant were those related to the Golgi, endosome, nucleoside processing, and protein conjugation including ubiquitination, consistent with the role of VPS9-domain containing proteins in Rab5 activation [40]. *VPS9D1* expression was itself higher in subjects with the variant than those without but failed to reach the FDR threshold. As noted above, *VPS9D1* expression was significantly higher in sepsis survivors than in sepsis non-survivors. This was also true of many RAS oncogene family members, including *RAB5C* (Additional file 2). The association of *VPS9D1* variants with differential gene expression and pathways which this gene is itself associated with supports the biological relevance of these variants.

### Mitochondrial gene associations

Given the metabolomic evidence of mitochondrial energetic dysfunction in sepsis death [11,41-43], genetic associations were sought between sepsis outcome and mRNA variants that mapped to mitochondrial genes in the germline and mitochondrial (mt) genome. Genotypes were determined for nucleotides in mitochondrial transcripts where at least 85% of the mitochondrial genome was represented at a sequence depth of >16-fold (reference allele, variant allele, heteroplasmy). Twenty sepsis non-survivors and 58 sepsis survivors met these criteria. The total number of variants per sample was similar between groups (38.0 variants per sepsis non-survivor, 33.6 per sepsis survivor, and 37.7 per SIRS survivor of which there were 13). The number of variants possibly associated with altered protein function was also similar between groups (7.5 per sepsis non-survivor, 8.5 per sepsis survivor, and 9.6 per SIRS survivor). There were no significant differences in the presence of rare alleles (MAF <1%) per sample between groups, nor in the number of variants per gene. We also looked at MT haplogroups and sub-haplogroups focusing specifically on haplogroup H and the MT-*ND1* T4216C variant, which have previously been associated with sepsis survival [44,45]. Using the HaploGrep online tool [46], we observed a similar haplogroup H frequency in sepsis survivors (47.2%) and non-survivors (45.8%). Likewise, no differences in MT-*ND1* T4216C variant frequency were observed.

Maternally-inherited mitochondria are not a uniform population. Moreover, mitochondria are prone to a high mutation rate. As a result, there is heterogeneity in the mitochondrial population at the cell and organism levels, known as heteroplasmy. Heteroplasmy has the potential to mitigate or aggravate mitochondrial disease-associated mutations depending on the representation of affected mitochondria in relevant tissues [47]. We hypothesized that heteroplasmy may be associated with sepsis non-survival. We therefore measured the frequency and pattern of heteroplasmy in the complete mitochondrial genome in sepsis survivors compared to sepsis non-survivors. This was determined by variant read counts followed by data visualization in Integrated Genomics Viewer. No difference between sepsis non-survivors and sepsis survivors was identified. In addition, a more stringent analysis of 41 well-characterized points of heteroplasmy [48,49] revealed no significant differences between sepsis survivors and non-survivors. The sensitivity of these genetic comparisons, however, was greatly limited by sample size.

## Discussion

This analysis of peripheral blood mRNA sequences revealed key genes, pathways, and genetic variants associated with SIRS, sepsis survival, and sepsis non-survival. Sepsis (SIRS due to infection) was distinguished from SIRS (without infection) by increased expression of many genes involved in the immune and defense response, vesicle biology, and apoptosis. A similar increase in gene expression was observed in sepsis survivors compared to sepsis non-survivors, particularly interferon γ-induced genes, immune and defense response, cytokine pathways, antigen processing and presentation, and protein kinase signaling. Moreover, expressed sequence variants in *VPS9D1* were significantly associated with sepsis outcomes.

Understanding host response to sepsis and how it differs from a non-infectious SIRS illness has been a major focus of research for some time. Likewise, great efforts have been made to identify host factors associated with sepsis recovery versus death. In recent years, tools have become available to explore these questions comprehensively including gene expression analysis [50-53], metabolomics [11,54,55], proteomics [11,56-58], microRNA analysis [59-61], as well as the integration of these multi-omic approaches with comprehensive clinical features [11]. In contrast to previous work, this study utilized mRNA sequencing, rather than microarrays, to characterize the transcriptome. In doing so, we confirmed the importance of key biological pathways both in the successful response to sepsis, which was observed to be absent in SIRS without infection and muted in sepsis non-survivors. The use of mRNA sequencing to define the transcriptome also enabled the identification of expressed, potentially function-affecting, nucleotide variants associated with sepsis outcomes as well as an examination of allelic imbalance associated with those variants. To our knowledge, applying this approach to sepsis is novel in humans.

Expression analysis identified many genes involved in immune activation among sepsis survivors. Compared to sepsis survivors, subjects with SIRS and sepsis non-survivors both demonstrated decreased activation of these immune function-related genes. This muted response in SIRS was not unexpected given the absence of infection. However, the decreased representation of immune response in sepsis non-survivors suggested an ineffective or maladaptive host response to infection supporting previous observations that late phases of sepsis are characterized by a higher microbiological burden and death rate [62]. Interestingly, sepsis survivors were also distinguished by increased expression of genes related to the mammalian target of rapamycin (mTOR) pathway and autophagy - a mechanism critical for organelle and mitochondrial recycling as well as selective intracellular degradation of invading pathogens [63]. Another notable pathway expressed at higher levels in sepsis survivors related to the receptor for advanced glycation endproducts (RAGE) pathway and included the RAGE-related genes *S100A8*, *S100A9*, *S100A12*, and formyl peptide receptor 1 *(FPR1)*. S100A8 and S100A9 are important in NLRP3-inflammasome activation [64]. Supporting the significance of the inflammasome in sepsis survivors, they also exhibited increased expression of genes downstream from inflammasome activation including interleukin-1 receptor 2 (*IL1R2*), *IL18R1*, and the IL-18 receptor accessory protein (*IL18RAP*).

Assuming a rare variant - common phenotype hypothesis, expressed nucleotide variants were sought that showed an association with sepsis survival. Potentially functional variants in Vacuolar Protein Sorting 9 Domain-containing gene 1 (*VPS9D1*) were associated with sepsis outcome. *VPS9D1*, whose expression was significantly higher in survivors compared to non-survivors, encodes a VPS9 domain-containing protein with ATP synthase and GTPase activator activity [65]. VPS9 domains are highly conserved activators of Rab5 GTPase which regulates cell signaling through endocytosis of intracellular receptors [40]. Nine non-synonymous substitutions were identified in *VPS9D1*. The most common *VPS9D1* missense variant, p.Thr486Pro, was located in the VPS9 domain. VPS9D1 has also been shown to interact with GRB2 (growth factor receptor-bound factor 2) [66], which was also more highly expressed in sepsis survivors and in those with *VPS9D1* variants. In T-cells, GRB2 functions as an adaptor protein that binds SOS1 in response to growth factors [67]. This results in activation of membrane-bound Ras, promoting increased cell proliferation and survival. Moreover, GRB2 functions in calcium-regulated signaling in B-cells [68]. *GRB2* has an alternatively spliced transcript that encodes the GRB3-3 isoform. GRB3-3 lacks an SH2 domain which normally suppresses proliferative signals, and as a result, GRB3-3 activates apoptosis via a dominant-negative mechanism [69,70]. Both isoforms associate with heterogeneous nuclear ribonucleoprotein C and are modulated by poly(U) RNA in the nucleus, where they are felt to perform discrete functions [70]. Thus, upregulation of *VPS9D1* and concurrent *VPS9D1* missence variants, combined with upregulation of *GRB2* in sepsis survivors, presents a complex interaction that balances increased cellular proliferation and survival, B- and T-cell activation, and proapoptotic activity, all of which are key processes in sepsis.

It should be noted that gene expression changes described in this report are based on peripheral blood cells and may not reflect changes occurring at the tissue level such as liver and muscle which are important in sepsis outcomes [11]. Therefore, these findings should not be construed to represent the host’s response in its totality. Moreover, differences in gene expression between survivors and non-survivors could reflect a confounding, pre-morbid condition rather than sepsis-related biology, a hypothesis with precedent as it relates to long-term disability among sepsis survivors [71]. These concerns are not expected to impact expressed genetic variant identification since these are likely to be germline changes. However, it is possible that variants in genes expressed at a low level might escape our detection due to inadequate coverage. Additional studies are therefore needed to clarify the relationships between these variants and the survival/death molecular phenotypes. Specifically, these associations require replication in several, larger cohorts containing patients from more homogeneous genetic backgrounds. Subjects were selected for analysis primarily based on sepsis diagnosis, severity, and outcome, which introduces the possibility of selection bias and underscores the need for validation in independent populations. In addition, the functional consequences of the *VPS9D1* missense variants should be ascertained.

## Conclusions

The CAPSOD cohort is an ethnically, demographically, and clinically diverse population of subjects with early, community-onset sepsis. In addition to clinical phenotyping, this population has been characterized at the molecular level including proteomics, metabolomics [11], and now transcriptomics using RNA sequencing. Blood proteomics and metabolomics highlighted the changes occurring at the system level whereas transcriptomics largely reflected immune cell activity. We identified a more robust immune response in sepsis as compared to SIRS which was muted in sepsis non-survivors, even when considering a 28-day mortality endpoint. Genes encoding expressed sequence variants that associated with sepsis outcomes were sought. No statistically significant variants in mitochondrial genes or in mitochondrial heteroplasmy were identified. However, *VPS9D1* contained variants that were significantly more likely to occur in sepsis survivors. Variants in *VPS9D1* were themselves associated with altered gene expression, affecting biological pathways which VPS9D1 plays a known or putative role. This research confirms prior findings implicating immune response as important in the sepsis response. It also identifies genetic variation in two genes, not previously implicated in sepsis, that play potentially important roles in determining sepsis outcome.

## Additional files

Additional file 1:
**Location of VPS9D1 missense variants.** Data presented in Table 2 and in the manuscript are based on the bolded transcripts. Nomenclature is based on Human Genome Variation Society guidelines. Additional file 2:
**List of all statistically significant differentially expressed genes between SIRS, Sepsis Survivor, and Sepsis Non-survivor.** Values presented are counts log2 (x + 1). * denotes significantly different from SIRS. # denotes significantly different from Sepsis Survivor. Yellow-highlighted cells indicate unannotated genes. Additional file 3:
**Gene list enrichment analysis and candidate gene prioritization based on functional annotations for clinical categories.** Top 50 pathways for each comparison are presented. Comparisons include SIRS vs. Sepsis (including survivors and non-survivors), SIRS vs. Sepsis Survivors, and Sepsis Survivors vs. Sepsis Non-survivors. Additional file 4:
**Gene list enrichment analysis and candidate gene prioritization based on functional annotations for** ***VPS9D1*** **variants.** Top 50 pathways are presented for genes differentially expressed between subjects with sepsis who had a *VPS9D1* variant (n = 36) and those without (n = 64). Subjects with inadequate sequencing coverage across the variant were removed from this analysis.

## Abbreviations

ANOVA: Analysis of variance
APACHE II: Acute physiology and chronic health evaluation II
CAPSOD: Community acquired pneumonia and sepsis outcome diagnostics
CPGM: Center for pediatric genomic medicine
ED: Emergency department
FDR: False discovery rate
GATK: Genome analysis tool kit
RUNES: Rapid understanding of nucleotide variant effect software
SIRS: Systemic inflammatory response syndrome
SNP: Single nucleotide polymorphism
VCF: Variant calling file
